# *Escherichia coli* Group 2 capsules and their interplay with bacteriophages

**DOI:** 10.3389/fmicb.2025.1588121

**Published:** 2025-09-18

**Authors:** Naoise McGarry, Catherine Toner-Bartelds, Stephen G. J. Smith

**Affiliations:** Department of Clinical Microbiology, School of Medicine, Trinity College, Dublin, Ireland

**Keywords:** *Escherichia coli*, ExPEC, bacteriophage, capsule, lipopolysaccharide, LPS, polysaccharides, phage

## Abstract

**Introduction:**

Extracellular polysaccharide capsules of Gram-negative bacteria, such as *Escherichia coli (E. coli)*, mediate interactions with host defences and bacteriophages (phages). Capsules may act as barriers to infection or serve as essential receptors when phages rely on capsule recognition and degradation by depolymerases.

**Methods:**

In this study, we examined the Group 2 K2 capsule of the extra-intestinal pathogenic *E. coli* (ExPEC) prototype CFT073 to determine its role in phage infection. We assessed whether the capsule acts as a barrier or receptor and explored the effect of temperature on such interactions. Additionally, we analysed *E. coli* genomes to identify whether capsule biosynthesis genes were co-located with other loci associated with phage defence. The evolutionary context of these associations was alsoc explored.

**Results:**

The K2 capsule of CFT073 exhibited dual functionality, acting both as a barrier to phage infection and as a receptor facilitating infection. A previously unrecognized synergy was observed between capsule expression and a type IV toxin–antitoxin (TA) system in CFT073. It was also shown that co-localisation of capsule and TA loci was present in more than 500 *E. coli* genomes, indicating a conserved association. Further, these systems were shown to be horizontally co-acquired on a common pathogenicity island.

**Discussion:**

These findings highlight the complex role of capsules in phage interactions and suggest that their functional linkage with TA systems may enhance bacterial persistence. The conserved co-acquisition of these loci on pathogenicity islands underscores their potential importance in the evolution and success of ExPEC pathogens.

## Introduction

Extra-intestinal pathogenic *Escherichia coli* (ExPEC) is a collective term for the pathogenic strains of *E. coli*, which are responsible for extra-intestinal disease, including meningitis, urinary tract infections (UTIs), and bloodstream infections (Dale and Woodford, 2015). ExPEC possesses genes encoding specialised virulence factors that enable the colonisation of the bacteria at the extra-intestinal sites, in addition to evasion of the host immune responses (Sarowska et al., 2019). Key virulence factors implicated in serum resistance and complement evasion during bloodstream infections include extracellular polysaccharides such as colanic acid, the O antigen moiety of lipopolysaccharide (LPS), and the K capsules, such as K1 and K2, which often function as molecular mimics to host molecules (Devine and Roberts, 1994; Buckles et al., 2009; Cross et al., 1986; Miajlovic et al., 2014).

Bacteriophage (phage) are viruses that can infect bacteria, often—but not always—resulting in bacterial cell death/lysis (Korf et al., 2019). However, the host range of the phage is generally narrow (Maffei et al., 2021). The limited host range is determined by a variety of host resistance mechanisms against phage, such as inhibition of phage receptor binding, restriction–modification systems, and toxin–antitoxin (TA) systems (Kelly et al., 2023). Such phage defence systems are almost always carried on horizontally acquired mobile genetic elements, such as prophage, transposons, and conjugative elements (Hochhauser et al., 2023). Moreover, phages, as with other viruses, require highly specific host surface receptors (and co-receptors) for adsorption and infection, which also results in the narrow host range (Korf et al., 2019). The specificity of phages to specific bacterial species and strains makes these viruses to be an attractive, alternative therapeutic strategy to antibiotics that are largely broad-spectrum in nature (Gorzynski et al., 2023). Extracellular polysaccharides and surface proteins are often act as phage targets. For example, phages that infect Gram-negative bacteria often target specific LPS moieties as receptors or co-receptors, in addition to outer membrane proteins (OMPs), pili, or capsule polysaccharides (Maffei et al., 2021; Battaglioli et al., 2011; Sarkar et al., 2014; Washizaki et al., 2016).

Phages that target O antigen moieties of LPS display a relatively narrow host specificity due to the variability of *E. coli* O antigens, with approximately 180 O types currently identified (Barbirz et al., 2008; Fratamico et al., 2016). Conversely, phages that target the core oligosaccharide constituent of LPS (such as P1) display a broader host specificity, as only five core types exist in *E. coli* (Amor et al., 2000; Broeker and Barbirz, 2017). *E. coli* capsules can also be common targets of phage (Kortright et al., 2020). *E. coli* Group 2 capsules K1, K5, and K29 (common among extra-intestinal isolates) have been specifically shown to be required for infection by several phage, including Bacteriophage 29 (which infects *E. coli* K29); K1E, K1H, and K1F (which infect *E. coli* K1); and K1-5 (which infects *E. coli* K1 and K5) (Fehmel et al., 1975; Leiman et al., 2007). However, often where capsule is required for phage susceptibility, phage-encoded capsule depolymerases were implicated in infection, thus indicating that perhaps some of the aforementioned phage require capsule to gain access to secondary receptors via capsule degradation (Knecht et al., 2019). *E. coli* capsules are not the only capsular polysaccharides targeted by phage; for instance, *Klebsiella pneumoniae* strains possessing a K2 capsule (serologically and chemically distinct from *E. coli* K2 but with a similar importance in virulence) are targeted by several lytic phage, with epitopes of K2 polysaccharide serving as an essential co-receptor (Dunstan et al., 2023). Occasionally, both capsule and LPS are required as co-receptors, for example, phage PNJ1809-36 and its host, *E. coli* DE058 (K1 serotype) (Gong et al., 2021).

*E. coli* capsules, in addition to occasionally serving as a target for phage, can act as a steric barrier to phage, protecting the surface proteins or polysaccharides that are used as phage receptors (i.e., OMPs and LPS core oligosaccharide) (Scholl et al., 2005). It has been shown that the K1 capsule, frequently associated with extra-intestinal *E. coli* isolates and neonatal meningitis, provides a steric barrier to infection by T7 phage, which recognises the LPS core moiety as a primary receptor (Kortright et al., 2020; Scholl et al., 2005). Enzyme-mediated degradation of the K1 capsule restored T7 phage susceptibility, thus proving that the mechanism of resistance to T7 in K1 *E. coli* occurs by virtue of steric hindrance (Scholl et al., 2005). Similarly, smooth LPS (LPS possessing an O antigen) has been shown to act as a barrier to infection by several phages, including T6, through preventing access to surface receptors such as OmpC, OmpF, Tsx, and OmpA (van der Ley et al., 1986). To overcome the *E. coli* extracellular capsule as a defence mechanism, many phages have evolved to possess the aforementioned capsule depolymerase enzymes with specificities for *E. coli* K antigens, which include K1, K3, K5, K7, K12, K20, K26, K28-1, K28-2, K30, K31, K32, K36, K38, K39, K42, and K95, and likely more (Knecht et al., 2019; Scholl et al., 2005). Depolymerases have also been identified in phages that target *Acinetobacter baumannii*, *Klebsiella pneumoniae*, and *Pseudomonas aeruginosa*, indicating that the capsule polysaccharides often encoded by these other pathogenic species also serve as passive barriers to phages (Dunstan et al., 2023; Glonti et al., 2010; Dunstan et al., 2021).

In addition to physical barriers, bacteria have acquired other mechanisms to evade infection by the phage. TA systems are ubiquitous systems encoded by adjacent genes, usually consisting of two or occasionally three components, which result in the production of a toxin that interferes with metabolism, and a cognate antitoxin (LeRoux and Laub, 2022). Toxins encoded by TA systems can reduce metabolism in diverse ways, for example, by damaging the cell membrane, which reduces ATP production, by modifying mRNA/tRNA/rRNA, which result in halted translation, and by interfering with replication through adenylation of replication enzymes, DNA gyrase and topoisomerase IV (Kelly et al., 2023). To exert these effects on bacterial cells, TA systems typically feature a protein toxin that inhibits host cell growth using one of the above mechanisms to result in eventual cell death, if not counteracted by the cognate antitoxin (LeRoux and Laub, 2022). Under normal conditions, the antitoxin is constitutively expressed, which prevents toxin activity, allowing normal metabolism and growth. However, upon encountering stress, such as exposure to bacteriophage, antibiotics, or AMPs, the antitoxin is degraded, permitting the inhibitory effect of the toxin. However, toxin activity may lead to growth arrest, persistence, or programmed cell death. As such, TA systems provide a selective advantage to bacteria by promoting survival in harsh environments or by preventing widespread phage infection and propagation via altruistic cell ‘suicide’ (also known as abortive infection) (Kelly et al., 2023).

Specific serotypes of the aforementioned O antigen polysaccharide and polysaccharide capsules implicated in *E. coli* complement evasion and bloodstream infections are correlated with ExPEC infections (Lupo et al., 2021; Ciesielczuk et al., 2016). Strain CFT073 (serotype O6:K2:H1) is a prototypic urosepsis isolate utilised by this laboratory (and others) to study serum resistance and UTI pathogenesis (Buckles et al., 2009; Miajlovic et al., 2014; Battaglioli et al., 2011; Sarkar et al., 2014; Smith et al., 2010; Hryckowian et al., 2015; Hryckowian and Welch, 2013; Snyder et al., 2004). The extracellular polysaccharides O6 antigen and the K2 capsule have been specifically implicated in serum resistance but may also serve as receptors or barriers to phage (Buckles et al., 2009; Sarkar et al., 2014). Understanding the interactions of CFT073 with bacteriophage may provide insight into the phage defence systems of ExPEC, as well as highlight the phage (or phage-derived enzymes), which may be explored for therapeutic use to control disseminated infections. As such, the interactions of CFT073 with several bacteriophage were the focus of this study.

## Materials and methods

### Bacterial strains, phage, plasmids, and culture conditions

The bacterial strains and plasmids utilised in this study are listed in Supplementary Tables S1, S2. Bacteria were grown overnight (approximately 18 h) in Lysogeny Broth (LB) NaCl, 5 g/L, Tryptone, 10 g/L Yeast Extract, 5 g/L, or on LB agar (Sigma) at 37 °C, 30 °C, or 20 °C, where indicated. Where required, antibiotics were added to growth media at the following concentrations: 10 μg/ml, gentamicin; 50 μg/ml, kanamycin; and 100 μg/ml, carbenicillin (all Sigma).

### Mutagenesis

Mutants were constructed as per the Lambda (*λ*)-Red recombination protocol, as detailed by Datsenko and Wanner (2000). Kanamycin (pKD4) or Gentamicin (pMH2) resistance cassettes were amplified through PCR by primers that had been designed to contain homologous flanking sequences to the target genes. Amplicons were purified using the Monarch DNA and PCR Cleanup Kit (New England Biolabs) and precipitated as per the Co-Precipitant Pink (Bioline) protocol before resuspension in 4 μl molecular-grade water (Thermo Fisher). The λ-red recombination genes on the pKD46 vector were induced through the addition of l-arabinose (final concentration 10 mM, Sigma) to CFT073/pKD46 cultures for 1.5 h at 30 °C. The temperature-sensitive pKD46 plasmid was removed from CFT073 through incubation at 37 °C. Putative mutants were confirmed through PCR. All mutant strains are listed in Supplementary Table S1. Plasmid vectors used to amplify antibiotic resistance cassettes for mutagenesis are listed in Supplementary Table S2. All oligonucleotides used for recombination and mutant screens are listed in Supplementary Table S3. To remove antibiotic resistance cassettes, mutants were transformed with pCP20, which possesses the yeast *flp* (flippase) gene (FLP), resulting in a marker-less mutant. The protocol followed for FLP-mediated cassette removal is detailed in Datsenko and Wanner (2000). A double mutant deficient in the AbiEi system and the *kslCDABE* operon was created by preparing a Δ*ksCDABE* lysate using φEB-49 and transducing into the ΔTA mutant.

### ELISA

Cultures were washed two times in PBS and standardised to OD_600nm_ = 0.1 in PBS before plating 25 μl onto a poly-l-lysine-treated 96-well plate (Greiner) for overnight incubation at 4 °C. The plates were centrifuged for 1 min at 10,000×*g* before the removal of the supernatant and the addition of 100 μl of 0.1% v/v glutaraldehyde (Sigma) for 10 min at room temperature (RT). Wells were washed three times with PBS-T before the addition of 200 μl 5% (w/v) skimmed milk (Marvel) in PBS-T. Plates were blocked for 1 h at 37 °C before the addition of anti-OmpF or anti-K2 antibody diluted at a ratio of 1:1,000 to each well for incubation at 37 °C for 1 h. Wells were washed three times vigorously with PBS-T before the addition of 200 μl anti-rabbit-AP antibody (Cell Signalling Technologies) at a ratio of 1:10,000 dilution to each well. Wells were washed for a final three times with PBS-T before adding 100 μl of the 1-Step PNPP (Thermo Fisher) to each well. The substrate was mixed thoroughly by gently agitating the plate at 20 °C for 30 min. To stop the reaction, 50 μl 2 M NaOH was added. Absorbance was measured at 405 nm.

### Western blot

To prepare whole-cell lysates for SDS-PAGE, 1 mL of overnight or exponential cultures of CFT073 strains was centrifuged at 13,000*g* and resuspended in 1X Laemelli (Sigma) to a final concentration of 10 OD_600nm_. Samples were boiled at 100 °C for 10 min and allowed to cool before adding 20 μg/ml Proteinase K (Sigma) and incubating at 56 °C for 1 h. A total of 20 μl of samples were stored at −20 °C or run on a 4–20% TruPAGE Precast gel at 180 V in 1X TruPAGE SDS Buffer (both Sigma). Following electrophoresis, gels were transferred onto polyvinyl difluoride (PVDF) using the iBlot 2 (Thermo Fisher) for dry transfer. Membranes were blocked in 5% bovine serum albumin (BSA; Sigma) in 1X PBS supplemented with 0.1% Tween 20 (0.1% PBS-T) for 1 h at RT. Membranes were incubated in anti-O6 or anti-K2 primary antibodies (Statens Serum Institut) diluted at a ratio of 1:500 in 5% BSA 0.1% PBS-T for 90 min at RT. Membranes were washed three times for 10 min in 0.1% PBS-T before incubating with anti-rabbit-HRP antibodies (Cell Signalling Technologies) diluted at a ratio of 1:10,000 in 5% BSA 0.1% PBS-T for 45 min at RT. Membranes were washed four times before staining for 5 min with Pierce ECL Western Blotting Substrate (Thermo Fisher), as per the manufacturer’s protocol. Membranes were imaged under the ImageQuant Las4000 (GE Healthcare Life Sciences). ImageStudio Lite (v 5.5.4) was utilised to relatively (no absolute values) quantify bands from the Western blot (TIFF images). The quantitative values reflect the relative amount of polysaccharide as a ratio of each band relative to the gel background.

### RNA extraction and quantification

RNA from cultures grown at 20 °C, 30 °C, or 37 °C was extracted as per the Monarch Total RNA Miniprep kit protocol (New England Biolabs). RNA was eluted in 30 μl nuclease-free water (Millipore), and quality and concentration were verified using the Qubit and NanoDrop analysis instruments (Thermo Fisher). Real-time quantitative PCR (RT-qPCR) was used to quantify gene expression in *E coli*. The Luna® Universal One-Step RT-qPCR Kit was used for all RT-qPCR reactions in 20 μl volumes with 10 ng RNA as template. Standard curves were generated with fixed twofold serial dilutions of wild-type CFT073 RNA. RT-qPCR reactions were set up in triplicate in MicroAmp Fast Optical 96-well reaction plates (Applied Biosystems) and run on the StepOnePlus Real-Time PCR System (Thermo Fisher). Default ‘Fast’ RT-qPCR and melt curve settings were utilised. All oligonucleotides used for RT-qPCR are listed in Supplementary Table S3. Data analysis was carried out using the StepOne software or Prism GraphPad. Housekeeping gene *rplT* was used as an internal control. Relative expression (or ‘RQ’) is calculated automatically by the instrument. RQ represents the fold change compared to the calibrator sample. The calibrator sample has an RQ value of 1, and the values for all other samples are expressed as fold-change relative to the calibrator.

### Phage titration

Bacteriophages utilised in this study are listed in Supplementary Table S4. Warm top agar was prepared by adding 0.7% agar (Sigma) to 100 mL LB broth, followed by sterilisation in an autoclave. Top agar was maintained in a molten phase by storing it in a 56 °C water bath. Bacteria were grown overnight and standardised to OD_600nm_ = 0.4 before 100 μl culture was added to 6 mL top agar and poured atop solid, standard LB agar plates (with appropriate selection if required). Plates with top agar were allowed to sit lid-side up for 20 min before serial dilutions of bacteriophage were spot-titered (10 μl) onto the plates. The plates were incubated at 20 °C or 37 °C upright overnight, followed by calculation of PFU/ ml after 18 h. PFU/ml = plaques × dilution factor/volume plated in ml. Experiments were conducted in triplicate to conduct statistical analysis.

### Bioinformatics

The CFT073-encoded TA sequence (CDS c3681–c3682) (1,306 bp) was input to the NCBI BLAST alignment search tool with the specifications to search within *Escherichia coli* (*E. coli*) only, yielding 557 results. Following duplicate removal and removal of sequences with low query cover (<95%), 502 *E. coli* assemblies possessing genes homologous to c3681/2 with distinct accession numbers were analysed to determine the context of the TA system genes, i.e., to determine whether the genes were located upstream or downstream of capsule genes, as in CFT073. The distance between c3682 and *kpsF* was determined for the assemblies by (i) performing a nucleotide blast (blastn) of c3682 and *kpsF* against the 502 FASTA files of interest in the directory, (ii) extracting the coordinates for each ‘hit’, and then (iii) calculating the distance between the two coordinates. The assemblies were further examined to explore the phylotype and serotypes. Phylogroups were assigned via ‘ezclermont’ version (v) 0.6.3 (Waters et al., 2020), sequence types were identified using ‘mlst’ v2.23.0, and serotype (O and H) were identified using ‘ectyper’ v1.0.0 (Bessonov et al., 2021). Finally, capsule ‘K’ serotypes were identified using fastKaptive v0.2.2, a blast-based *in silico* typing tool (Holt et al., 2020). K locus genome sequences where K serotype had been phenotypically confirmed were used as input for fastKaptive to allow for accurate interpretation of the output. The results from the above analyses were collated and exported to Excel and statistically analysed using Fisher’s exact test, or the N1 Chi-square test via R, where stated. The assembled genomes were also uploaded to Ridom SeqSphere v10 to produce SNV and SNP analyses, core genome multilocus sequence typing (cgMLST), as well as a phylogenetic tree showing the relatedness of 100 of the assemblies. Gene synteny was visualised by extracting a Genbank file of the 50 kb region of interest for each genome analysed and subjecting a random cohort to ‘clinker’ cluster analysis, clinker v0.0.029 (Gilchrist and Chooi, 2021). To control for biases in the dataset used in this study, a collection of ExPEC genomes (BSAC2 collection) was utilised to explore the prevalence of the TA system in infection-associated ExPEC assemblies. In short, the fastq files from PRJEB44839 were retrieved from the European Nucleotide Archive (ENA) and assembled using Unicycler v0.5.1 before blastn analysis to determine the presence or absence of the TA system in the assemblies (Pöntinen et al., 2024). Assemblies with and without the TA system were analysed as above, to check serotype, phylogroup, and sequence type.

### Statistical analyses

Statistical analyses conducted are indicated in the relevant figure legends. All statistical analysis was carried out using the GraphPad Prism software unless otherwise stated. In all data, a *p* value of ≤ 0.05 is denoted *; a *p* value of ≤ 0.01 is denoted **; a *p* value of ≤ 0.001 is denoted ***; and a *p* value of ≤ 0.0001 is denoted ****.

## Results

### O6 antigen is essential for infection by several *Siphoviridae*

To determine whether the extracellular glycome of prototypic urosepsis isolate CFT073 was implicated in infection by φEB-5, φEB-32, φEB-47, or φEB-49, strain CFT073 wild-type and a series of extracellular polysaccharide mutants were subject to phage titration assays conducted at 20 °C (Battaglioli et al., 2011). Strikingly, in comparison to the wild-type control plate, on which there was total lysis by the phage, the O antigen-deficient strains (strains deficient in *waaL, waaG*, or *wzy*) displayed confluent growth, indicating resistance to phage infection. Interestingly, the K2 capsule mutant Δ*ksl* was completely lysed by the cohort of EB phage at levels comparable with wild-type, demonstrating that the K2 capsule is not a receptor or co-receptor for infection by these phage. These data indicate that the O6-specific O antigen side chain of ExPEC is targeted by transducing *Siphoviridae* φEB-5, φEB-32, φEB-47, and φEB-49. This hypothesis was confirmed, as through restoration of full-length LPS, by complementing *waaG* and *wzy* mutants with (i) pBAD-His-WaaG (Muheim et al., 2016) and (ii) pBWB536, which rendered the bacteria sensitive to EB phage at wild-type levels (see Table 1).

**Table 1:** O6 antigen is a target for several transducing bacteriophage of CFT073.

Strain	φEB-5	φEB-32	φEB-47	φEB-49
CFT073	1.20 × 10^9^	2.40 × 10^9^	1.65 × 10^9^	2.00 × 10^9^
Δ*ksl*	2.34 × 10^9^	2.28 × 10^9^	2.21 × 10^9^	2.12 × 10^9^
Δ*waaL*	**0**	**0**	**0**	**0**
Δ*wzy*	**0**	**0**	**0**	**0**
Δ*ksl* Δ*waaL*	**0**	**0**	**0**	**0**
Δ*waaG*	**0**	**0**	**0**	**0**
Δ*waaG* pBAD-His-WaaG	1.38 × 10^9^	2.24 × 10^9^	1.87 × 10^9^	1.68 × 10^9^
Δ*wzy* pBWB536	1.81 × 10^9^	1.45 × 10^9^	1.70 × 10^9^	1.28 × 10^9^

Serial dilutions of φEB phage lysates were spot-titered onto CFT073 strains. Plates were incubated lid-up at 20 °C overnight. Average PFU/ml displayed (*N* = 4).

### K2 capsule expression at 37 °C blocks infection by transducing *Siphoviridae*

As shown above, several *Siphoviridae* lyse O6-antigen possessing ExPEC at an optimal temperature of 20 °C, with φEB-5, φEB-32, φEB-47, and φEB-49 possessing little infectivity towards CFT073 at 37 °C (Battaglioli et al., 2011). To determine whether the K2 capsule, which is expressed at high levels during exponential and stationary phase growth at 37 °C, acts as a steric barrier to the φEB phage activity at this temperature, a *kslABCDE* mutant deficient in K2 capsule was incubated with serial dilutions of φEB-49 at 20 °C and 37 °C. These results (depicted in Table 2) showed that there was significantly increased infectivity of *ksl* mutants compared with the wild-type at 37 °C (*p* = 0.0003) and with the trans-complemented mutant Δ*ksl* pXLW36 (*p* = 0.0001). The capsule mutant was also significantly more sensitive to the other EB phage (data not shown). As a result, it can be assumed that the K2 capsule is not required for the *Siphoviridae* infection of ExPEC and instead hinders infection, likely through preventing access to the O6 antigen at 37 °C.

**Table 2:** K2 capsule is a barrier for φEB-49.

Strain	20 °C	37 °C
CFT073	1.65 ± 0.15 × 10^9^	33
Δ*ksl*	1.50 ± 0.30 × 10^9^	7.00 ± 1.5 × 10^7^
Δ*ksl* pXLW36	1.42 ± 0.24 × 10^9^	0

Overnight cultures were added to cooled, molten top agar and poured atop LB agar prior to the addition of serially diluted φEB-49 phage. Plates were incubated at 20 °C and 37 °C. Average PFU/ml values were calculated. *N* = 3. Wild-type at 37 °C: 2 × PFU/ml titres of 0, 1 PFU/ml titre of 100 (i.e., 1 colony on undiluted 10 μl spot titration). Statistical significance calculated via a one-way ANOVA and Dunnett’s multiple comparisons.

### K2 capsule is optimally expressed at 37 °C

**Figure 1:**
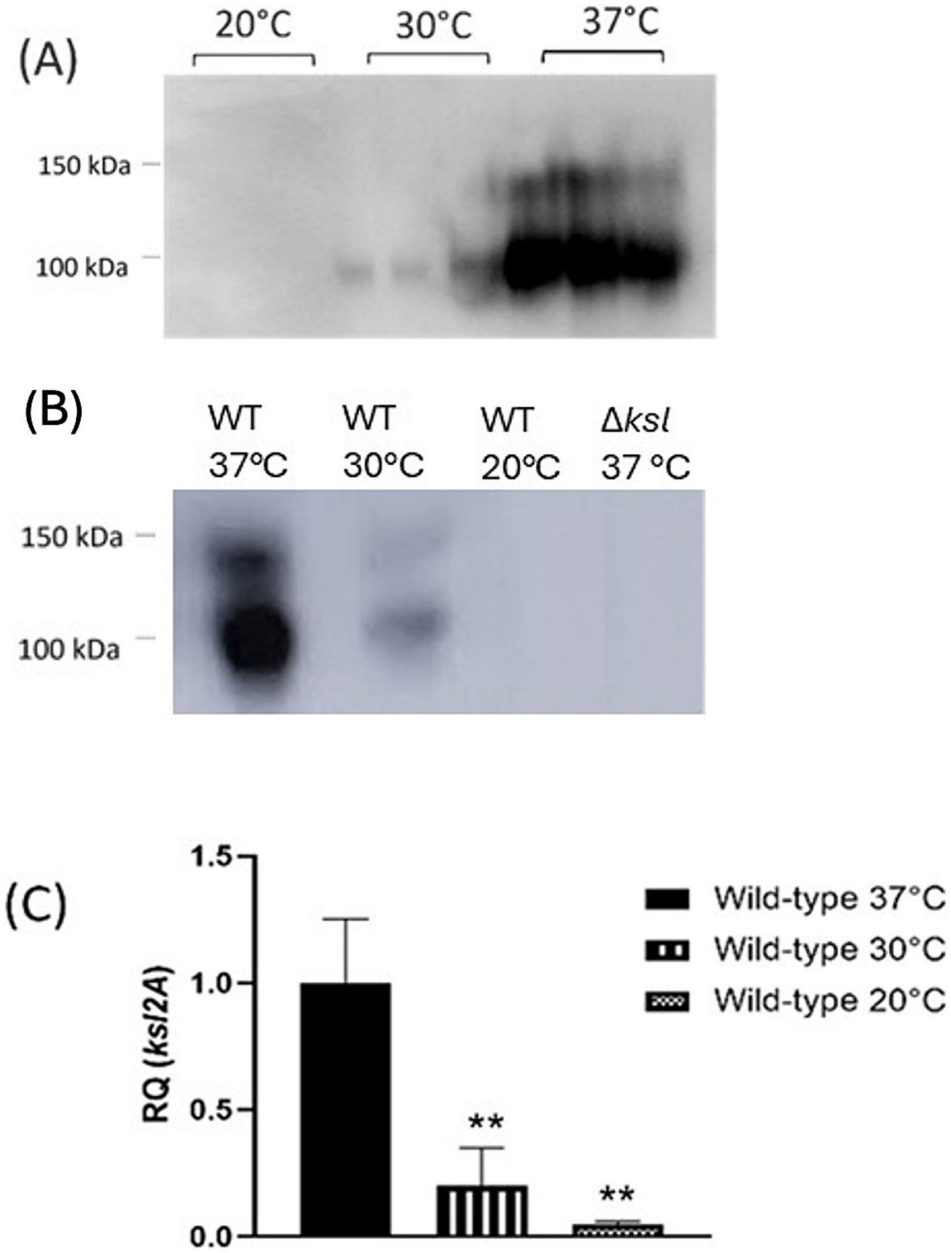
The *E. coli* K2 capsule is temperature-regulated. Cultures of CFT073 wild-type grown at 20 °C, 30 °C, and 37 °C were subject to **(A)** and **(B)** Western blot analysis of the K2 capsule, and **(C)** RT-qPCR analysis of *ksl2A* transcript levels to quantify relative transcription of the K2 capsule gene cluster. RQ = relative quantification. Statistical analysis conducted by One-way ANOVA and Dunnett’s multiple comparisons. **p ≤ 0.01, ***p ≤ 0.001.

To further elucidate how capsule expression may influence phage sensitivity, the K2 capsule of strain CFT073 grown at varying temperatures was analysed by Western blot. Western blot analysis of cultures grown at 20 °C, 30 °C, or 37 °C showed distinct levels of capsule expression. At 37 °C, whole cell lysates of wild-type CFT073 displayed high levels of K2 capsule in comparison to cells grown at 30 °C, which display very low K2 levels. Interestingly, at 20 °C, wild-type CFT073 capsule was almost completely undetectable, as seen in Figure 1A. As these Western blots were conducted on whole cell lysates, it is unclear whether the low level of capsule expression detected at 20 and 30 °C reflect intracellular capsule that was produced but not exported, or whether the capsule was cell-associated (or both). To elucidate whether reductions in K2 include surface capsule and to quantify the reduced capsule observed at 30 °C relative to 37 °C, ELISA for the K2 capsule was performed on CFT073 wild-type and mutants. Interestingly, cells grown at 20 °C and 30 °C displayed significant reductions in detectable K2 capsule when compared with cultures grown at 37 °C (data not shown). A mutant deficient in *ksl2ABCDE* was also subject to Western blot analysis, as in Figure 1B, to show antibody specificity. To determine whether the change in detectable capsule was at a transcriptional level and did not occur post-transcriptionally, RT-qPCR was conducted on RNA extracted from stationary phase cultures at 20 °C, 30 °C, and 37 °C. Transcription of *ksl2A* occurs at a very low level at 20 °C (RQ = 0.05), with a 20-fold reduction in expression relative to 37 °C transcript levels (RQ = 1) (*p* = 0.001). Moreover, there was a fivefold reduction in *ksl2A* expression at 30 °C (RQ = 0.2) relative to 37 °C (*p* = 0.003). These results are depicted in Figure 1C. Taken together, these data confirm that, as has previously been demonstrated for Group 2 capsules such as K1 and K5, the K2 capsule is temperature regulated with maximum expression from the *kslCDABE* operon at 37 °C and respective 20- and 5-fold decreases in expression at 20 °C and 30 °C (Aldawood and Roberts, 2022; Xue et al., 2009; Rowe et al., 2000; Simpson et al., 1996).

### The K2 capsule is a steric barrier for T2 phage infection

In this laboratory, it has been previously observed that wild-type CFT073 possesses little-to-no infectivity by T2 phage, with PFU/ml values ranging from 0 to 1 × 10^1^. Upon identifying the K2 capsule as a barrier to the cohort of φEB phage, wild-type CFT073 and mutants deficient in O6 antigen and K2 capsule were spot-titered with T2 phage to determine whether the extracellular glycome contributes to T2 resistance. Interestingly, as shown in Table 3, K2 and O6 are steric barriers to T2, with these polysaccharides shielding the receptor OmpF from the phage. MG1655—a K–12 strain, possessing rough LPS and no capsule polysaccharide—was used as a control due to its known sensitivity to T2. Strikingly, of the CFT073 derivatives, the mutant deficient in both O6 and K2 displayed the highest susceptibility to T2 phage, approximately 1.8 × 10^6^ PFU/ml, which indicates that the O antigen and capsule act synergistically in providing a barrier to T2 phage receptor OmpF. Complementation of O6 and K2 using vectors pXLW36 (contains *kslCDABE*) and pBWB536 (contains O6 *wb* cluster) resulted in very little infectivity by T2, similar to wild-type levels.

**Table 3:** K2 and O6 antigen are barriers to T2.

Strain	Average T2 susceptibility
CFT073 wild-type	1.0 ± 0.5 × 10^1^
Δ*ksl*	1.5 ± 0.25 × 10^5^
Δ*wzy*	3.2 ± 0.8 × 10^4^
Δ*waaL*	4.0 ± 0.62 × 10^4^
Δ*ksl* Δ*waaL*	1.8 ± 0.23 × 10^6^
Δ*ksl* pXLW36	0
Δ*wzy* pBWB536	1.5 × 10^1^
MG1655	8.0 ± 0.78 × 10 ^7^

Serial dilutions of T2 lysate were spot-titered onto CFT073 wild-type, mutant derivatives, complemented strains, and K–12 control MG1655. Plates were incubated lid-up at 37 °C overnight. PFU/ml values were calculated (*N* = 3).

### CFT073 has a TA system located upstream of K2 capsule genes

Interestingly, it was observed that a TA system (Type IV, homologous to the AbiEi/AbiEii abortive infection system, locus tags c3681–c3682 in CFT073 accession AE014075.1) was located just upstream of the region 1 genes of the K2 capsule gene cluster (Supplementary Figure S1). It has previously been shown that TA systems are often located next to virulence or antimicrobial resistance (AMR) genes (Horesh et al., 2020). Thus, it was hypothesised that the TA system located upstream of K2 capsule genes in *E. coli* CFT073 served a biological function, which may be conserved in other members of the species, for example, in maintaining the location or integrity of the capsule genes in ExPEC lineages. Alternatively, the TA system may play a role in phage defence, as Group 2 capsules often do, which may be indicative of a synergistic relationship between the two systems and their co-location.

### Co-location of capsule genes and TA system is not limited to CFT073

**Figure 2:**
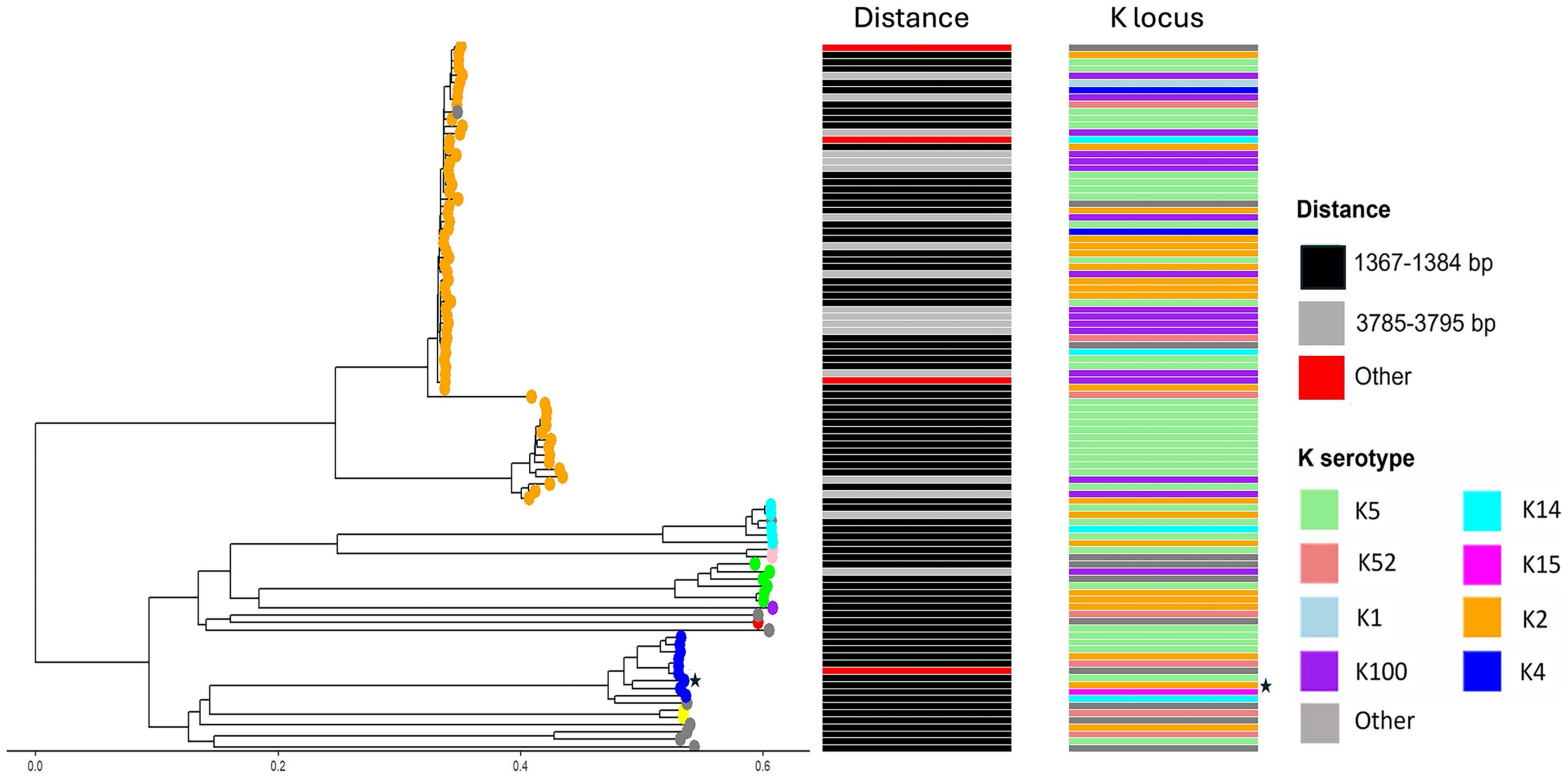
Capsule type and the distance between *kpsF* and the TA system are associated. The phylogenetic tree represents genetic relatedness between 100 random assemblies. The distance between *kpsF* and the TA system is coloured by colour mapping in column ‘Distance’, capsule serotype ‘K type’ is also depicted by colour mapping. Sequence types are represented as coloured tips on the phylogenetic tree, with orange corresponding to ST131, red to ST457, pink to ST393, cyan to ST69, yellow to ST12, green to ST38, purple to ST68, brown to ST405, and finally, blue to ST73. CFT073 (accession AE014075.1) is indicated with a black star. Graphical tree and colour mapping were made using ggtree and figtree on RStudio.

The sequence of c3681–c3682 (1,306 bp) was input into the NCBI BLAST alignment search tool with the specifications of search restricted to *E. coli* only, yielding 557 results. Following duplicate removal and removal of sequences with low query cover (<95%), 502 *E. coli* genomes possessing genes homologous to c3681/2 with distinct accession numbers were analysed to determine the context of the TA system genes, i.e., to determine whether the genes were located upstream or downstream of capsule genes. A phylogenetic tree representing the genetic relatedness of 100 random assemblies, as determined by core genome multilocus sequence typing (cgMLST), is depicted in Figure 2. The distance between c3682 and *kpsF* was determined for all 502 assemblies, with results depicted as a heatmap for the 100 plotted assemblies in Figure 2 and in the tabular form in Supplementary Table S5. This analysis showed that there are conserved distances between the chromosomal location of TA system genes and the capsule operon. Of the sequences analysed, 77% (385) of the genomes had a distance of 1,367–1,384 bp between the TA genes and the capsule operon, although the distance in majority of the 389 assemblies was 1,379 bp, indicating that perhaps the outliers within the group had acquired SNPs after the acquisition of the TA system, or the differences were due to errors in sequencing or assembly. Similarly, another conserved distance between the two genes was 3,785–3,795 bp, where 89 of the genomes possessed this distance. Moreover, 29 strains possessed the TA system at distinct distances from capsule genes, indicating that these bacteria likely did not co-acquire the two systems or that the region underwent recombination after co-acquisition.

The 502 genomes were subject to phylogenetic typing using the EzClermont (v0.6.3) tool. EzClermont is an *in-silico* method for using the Clermont 2013 PCR typing method, which assigns *E. coli* to specific phylogroups, A, B1, B2, C, Cryptic/unassigned, D E, F, and G (Clermont et al., 2013). Of the 502 sequences analysed, 415 (83%) belonged to the phylogroup B2, 69 (13%) belonged to phylogroup D, and 8 (1.6%) belonged to phylogroup F. Nine of the sequences could not be assigned to a specific phylogroup and thus were ‘unassigned’. The breakdown of assemblies into phylogroups is listed in Supplementary Table S5. Interestingly, phylogroups B2 and D are largely comprised of isolates associated with extra-intestinal infections, highlighting that most of the assemblies with the TA system belong to ExPEC-associated lineages (Chakraborty et al., 2015). Similarly, genomes were subject to analysis by MLST to determine the sequence type and, thus, genetic relatedness of the strains. Interestingly, 64% of the strains were of ST131 sequence type (*N* = 320). ST131 is the most prevalent sequence type amongst isolates associated with extraintestinal infections and is also correlated with multidrug resistant infections (Lee et al., 2010). The second most prevalent sequence type amongst strains with a TA system homologue was ST73 (*N* = 51 or 10%), the sequence type that includes prototypic urosepsis isolate CFT073. ST73, like ST131, is also a common sequence type found in disseminated extra-intestinal isolates and is often associated with higher virulence scores than other sequence types (Miajlovic et al., 2016). Eighteen strains belonged to ST69, another lineage associated with extra-intestinal infections (Riley, 2014). Sixteen strains belonged to ST405, whilst 11 of the strains belonged to ST393 and 12 to ST12, all frequently isolated from extra-intestinal infections (Lee et al., 2010). Forty-five of the strains were categorised as ‘Other’, as these sequence types are not well-defined, or were featured in low frequency (see Supplementary Table S6 for all sequence types identified by MLST).

Next, serotype finder (v2.0.1) and ectyper (v0.5.4) were used to determine O and H type (see Supplementary Table S5). Capsule type was then determined using fastKaptive (v0.2.2). The occurrence of specific capsule loci among the genomes is illustrated using the pie chart (Figure 2). Of the 502 assemblies analysed, the majority of the strains possessed Group 2 capsules. The majority of capsule types identified were those correlated with extra-intestinal infections, particularly K1, K2, K5, and K15 (Ciesielczuk et al., 2016). 178 (35.3%) of strains analysed with TA systems and capsule genes possess K5 capsules, with this capsule type being the most prevalent among those with c3682 homologues. The next most common capsule serotype was K2, with 108 (21.4%) of the strains possessing this K antigen. Seventy-six (15.1%) strains had K100 capsules, whilst 30 (5.9%) had K52 capsules and 16 (3.1%) had K14 capsules. Five strains had K4 capsules (<1%), three (<1%) strains had a K1 capsule, and a further two (<1%) had a K15 capsule. Of the 502 genomes, 85 strains (16.8%) possessed capsules that could not be typed as they did not match any of the reference loci used to interpret the fastKaptive output.

Interestingly, one of the K14 strains was an outlier in terms of distance between *kpsF* and c3682 (80,916 bp), suggesting that, perhaps in this strain, the TA system does not possess a relationship or association with capsule on a genomic island, or that these strains experienced an inversion/ transposition event after the co-acquisition of the genes. Another outlier (2,592 bp) represents the distance between the TA system and *kpsF* for one of the three K1 strains. The remaining two K1 strains had a distance of 1,378–1,379 bp between the two genes. The K100 strains possessed distances of 3,785–and 3,795 bp, indicating that, for these strains, the capsule genes and TA systems were co-acquired during a single horizontal gene transfer event, perhaps with SNPs accounting for the different distances between the systems in some strains. For 105 out of the 108 K2 strains, there was a distance of 1,379–1,384 bp between the c3682 and *kpsF* genes. Similarly, the K5 and K52 strains possessed distances of 1,377–1,379 bp. Analysis using the N1-Chi-squared test determined that there was a significant relationship between having a Group 2 capsule and a distance of 1,367–1,384 bp between the TA system and *kpsF* (*p* = 0.00017). Overall, these findings not only confirm a spatial link between capsule and the TA system across many *E. coli* genomes, but also indicate that the relationship between the two is specific to pathogens possessing Group 2 capsules, as well as the intergenic distance between c3682 homologues and *kpsF* being associated with the specific K type.

### Analysis of the local context of the TA system shows downstream synteny

**Figure 3:**
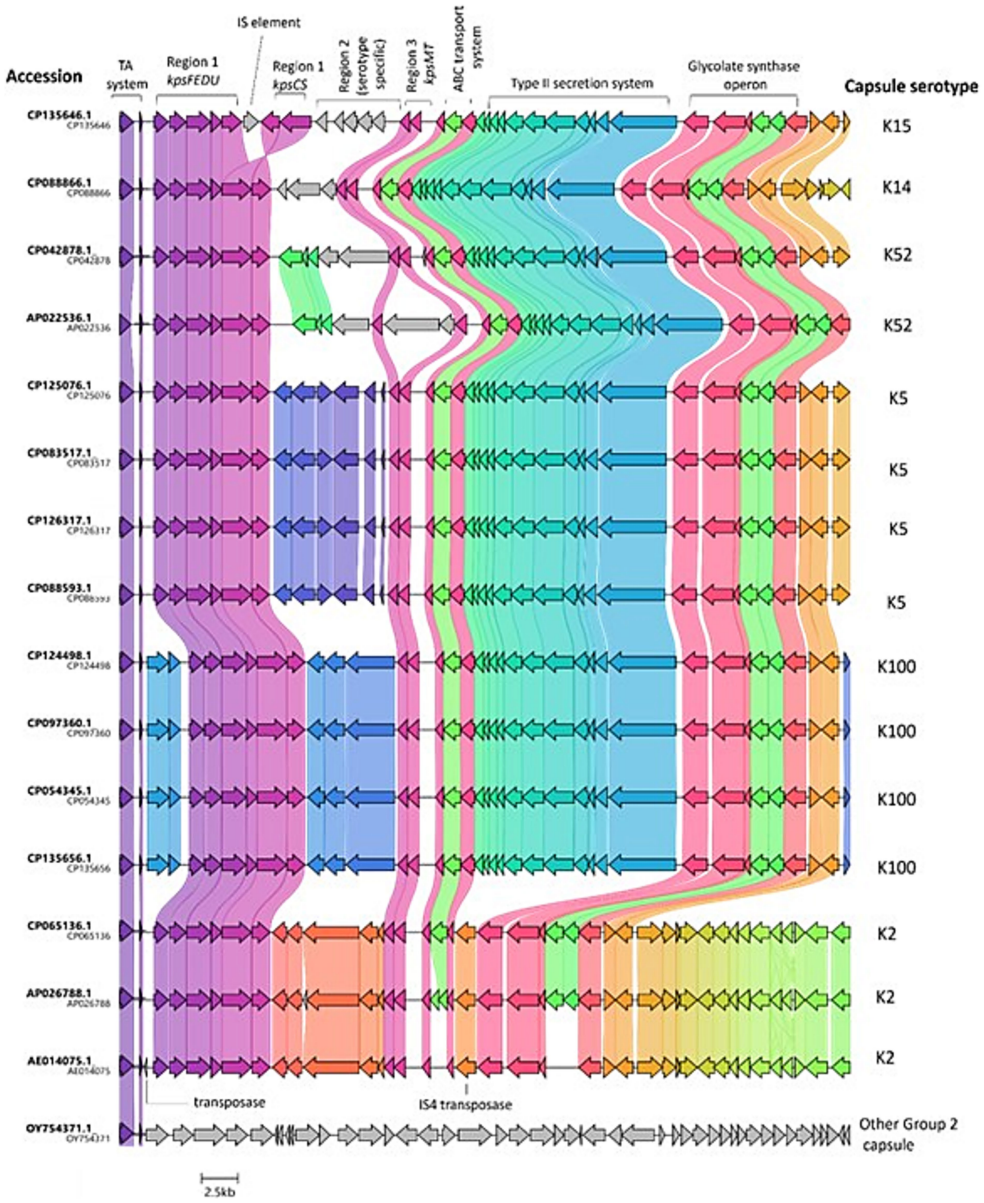
Local context of the TA system in *E. coli*. Figure depicts TA system, capsule, and additional downstream loci identified in a random sample of 16 of the analysed genomes, with the exception of OY754371.1 (outlier distance between *kpsF* and TA system) and AE014075.1 (CFT073; reference strain), which were added manually. The CDS are marked according to the annotated genomes. The genes are coloured based on group, with shading between strains indicating the level of similarity. The K antigen identified for each genetic capsule type is indicated in letters to the right, whilst the strain the capsule locus belongs to is indicated by letters to the left of the figure. GenBank files for the oriented 50 kb sequence of interest per strain were analysed via clinker v0.0.29 and exported to clustermap.js to visualise the synteny.

Next, the local context of the TA system in several of the analysed genomes was explored using the genome visualiser tool, Clinker (v0.0.29). As the distance between the TA and capsule genes appeared to be conserved irrespective of capsule serotype, it was hypothesised that perhaps the strains had acquired versions of a common pathogenicity island, which had undergone ancestral recombination/ further horizontal gene transfer, leading to the distinct changes in region 2 of the capsule genes. Visualisation of the 50 kb region, including the TA system and the capsule genes, showed high levels of synteny upstream and downstream of region 2 capsule genes. The most obvious differences between strains with regard to the local context of the TA system genes can be observed in Figure 3 in the regions corresponding to the region 2 capsule cluster, which will vary from serotype to serotype. Strains possessing the same capsule serotype display near-complete synteny for the entire 50 kb region. Interestingly, in strain CP093760.1 and the other K100 strains analysed, it appears that there was a further horizontal gene transfer or recombination event in an ancestral strain following the acquisition of the genes, which explains the longer distance between the TA system and *kpsF* in the K100 strains (3785–3,795 bp compared with 1,378 bp in strains with other capsule types). Additionally, the 50-kb region of interest varies slightly between the K2 strains examined in Figure 3 and the strains corresponding to K5, K14, K15, K52, and K100, as there appears to be a deletion (or lack of an insertion) in the region of interest downstream of the capsule cluster in the K2 strains. One of the 29 outliers in terms of distance between *kpsF* and the TA system was included in the synteny analysis, with the graphic in Figure 3 overleaf showing that, in strain OY754371.1, the TA system is not co-located with capsule genes and the region of interest does not display a synteny in the region of interest compared to the other genomes. However, taken together, the high levels of synteny regardless of strain or capsule serotype demonstrate that the capsule genes and TA system were likely co-acquired and exist on pathogenicity islands in many publically available *E. coli* genomes. This association between the TA system and capsule genes extends beyond the assemblies examined in this study, as 252/630 assemblies from the BSAC2 ExPEC collection possess the TA system upstream of the capsule gene cluster, with distances between the system and *kpsF* mirroring those previously observed for distinct capsule loci in this study (see Supplementary Figure S2 for graphical depiction and Supplementary Table S5).

### Mutagenesis of TA system genes in strain CFT073

To investigate the role (if any) of the TA system in CFT073 phage resistance, a TA mutant was made by disrupting c3681–c3682 by the *λ*-red system using a gentamicin cassette for gene replacement via homologous recombination, as demonstrated in Poteete et al. (2006).

### Mutants lacking capsule and TA display changes to phage susceptibility compared to strains lacking just TA or *kslCDABE* at 20 °C

A panel of seven phages that use LPS as a co-receptor for infection [selected from the BASEL (Bas) collection (Maffei et al., 2021)] were tested for their ability to infect CFT073 wild-type and mutant derivatives at both 20 °C and 37 °C, as well as a K-12 control (not shown). Additionally, the EB phage discussed above was also explored, but none of the mutants displayed changes in susceptibility to φEB-5, φEB-32, φEB-47, or φEB-49 (not shown).

At 20 °C, wild-type and ΔTA have a similar susceptibility to Bas phage, with no significant difference in the titres, with the exception of Bas 32, as shown in Table 4. The TA mutant was significantly less sensitive to Bas 32 than wild-type (*p* = 0.045), implying that perhaps the TA system affects the expression of the Bas 32 receptor. All of the strains tested, except the K-12 control, were resistant to Bas 35, indicating that CFT073 is either lacking in the receptor or that a component of the outer envelope, such as the O6 antigen, acts as a steric barrier to the receptor. Interestingly, the Δ*ksl* mutant and ΔTA Δ*ksl* are both resistant to Bas 04 and Bas 06, whilst the wild-type is sensitive, indicating that the K2 capsule is either the receptor or a co-receptor for these phages. Additionally, ΔTA Δ*ksl* is the only strain sensitive to Bas 46 at 20 °C, which indicates that perhaps both the TA system and the K2 capsule act as phage defence systems upon infection by Bas 46. Moreover, ΔTA Δ*ksl* is more sensitive to Bas 32 than the other strains, again suggesting that the TA system and capsule provide protection to Bas 32 at RT. Bas 09 infects wild-type and the TA mutant at comparable levels; however, the *ksl* mutant displays resistance to Bas 09, whilst the ΔTA Δ*ksl* mutant is more sensitive than wild-type and TA mutant. These results demonstrate that the K2 capsule is not the receptor for Bas 09 (due to ΔTA Δ*ksl* sensitivity); however, the TA system may act as a phage defence system in the absence of capsule, preventing infection of Δ*ksl* and leading to the increased sensitivity of the ΔTA Δ*ksl* mutant. Finally, Bas 07 infects all of the strains comparably, with the exception of ΔTA Δ*ksl*, which is resistant. This result raises questions over whether the AbiEi/AbiEii TA system and capsule interplay can increase susceptibility to some phage, or perhaps the absence of the TA system affects the expression of another TA system/receptor.

**Table 4:** Susceptibility of CFT073 and mutants to Bas phage at RT.

Phage	Wild-type	Δ*ksl*	ΔTA	ΔTA Δ*ksl*
Bas 04	3.0 ± 0.26 × 10^3^	0	2.5 ± 0.12 × 10^3^	0
Bas 06	2.5 ± 0.17 × 10^5^	0	2.2 ± 0.01 × 10^3^	0
Bas 07	1.5 ± 0.22 × 10^3^	1 ± 0.2 × 10^3^	1.3 ± 0.11 × 10^3^	0
Bas 09	1.3.0 × 10^3^	0	1.0 ± 0.3 × 10^3^	1.0 ± 0.08 × 10^4^
Bas 32	6.0 ± 0.44 × 10^5^	8 ± 0.16 × 10^5^	5.0 ± 0.14 × 10^3^	4.0 ± 0.13 × 10^7^
Bas 35	0	0	0	0
Bas 46	0	0	0	4.0 ± 0.77 × 10^3^

Stationary phase cultures were standardised and spot titered with Bas phage before incubating at 20 °C overnight. Average PFU/ml values were calculated and are displayed above. Statistical analysis was conducted by a two-way ANOVA and Tukey’s multiple comparisons. *N* = 3.

### Mutants lacking TA and capsule display changes to phage susceptibility at 37 °C

As observed at 20 °C, the K2 capsule appears to be a receptor or co-receptor for Bas 04 and Bas 06, with neither of the *ksl* mutants infected by these phage at either 20 °C or 37 °C (see Table 5). The *ksl* mutant was more susceptible to Bas 07 than wild-type and the TA mutant (although not significantly, *p* = 0.057 and *p* = 0.061), indicating that the K2 capsule hinders some Bas 07 activity at 37 °C. Again, the ΔTA Δ*ksl* mutant is more sensitive to Bas 32 at 37 °C than the other strains, further reinforcing that, for Bas 32 at 20 °C and 37 °C, the TA system and K2 capsule both serve as phage defence systems. Interestingly, at 37 °C, the TA system mutant was resistant to Bas 35, whilst the other strains were sensitive. All four strains were resistant to Bas 35 at 20 °C, indicating that the receptor is only expressed at 37 °C, and that the TA system permits infection by Bas 35, perhaps through capsule regulation, as the ΔTA Δ*ksl* mutant is more sensitive than the wild-type and TA mutant strains. Together, these findings indicate an interplay between the K2 capsule and the TA system in phage susceptibility, as well as in phage defence.

**Table 5:** Susceptibility of CFT073 and mutants to Bas phage at 37 °C.

Phage	Wild-type	Δ*ksl*	ΔTA	ΔTA Δ*ksl*
Bas 04	2.1 ± 0.18 × 10^3^	0	2.2 ± 0.09 × 10^3^	0
Bas 06	3.2 ± 0.44 × 10^3^	0	3.2 ± 0.3 × 10^3^	0
Bas 07	1.8 ± 0.23 × 10^3^	6.1 ± 0.6 × 10^4^	4.1 ± 0.16 × 10^3^	4.2 ± 0.12 × 10^3^
Bas 09	3.0 ± 0.18 × 10^3^	0	3.8 ± 0.6 × 10^3^	3.4 ± 0.11 × 10^3^
Bas 32	2.5 ± 0.08 × 10^4^	7.2 ± 0.65 × 10^5^	6.4 ± 0.08 × 10^4^	5.2 ± 0.91 × 10^6^
Bas 35	3.0 ± 0.7 × 10^3^	5.4 ± 0.42 × 10^3^	0	5.8 ± 0.6 × 10^4^
Bas 46	0	0	0	1.3 ± 0.16 × 10^3^

Stationary phase cultures were standardised and spot titered with Bas phage before incubating at 37 °C overnight. Average PFU/ml values were calculated and are displayed above. Statistical analysis was conducted by a two-way ANOVA and Tukey’s multiple comparisons. *N* = 3.

## Discussion

This study has identified the K2 capsule as a phage defence mechanism in ExPEC, although the polysaccharide also has other obvious roles in virulence, such as immune evasion and biofilm formation (Buckles et al., 2009; Valle et al., 2006; Goller and Seed, 2010). It was shown that the K2 capsule mutant derivatives of ExPEC prototype CFT073 display increased sensitivity to several transducing phages at 37 °C relative to wild-type, with regard to the φEB phage (*Siphoviridae*) and T2. As a result, it can be deduced that the K2 capsule is not a receptor or co-receptor in *Siphoviridae* infection of ExPEC, but, instead, hinders infection by preventing access to the O6 antigen (the target for EB phage). These results highlight the complexity of the roles played by glycome components O6 antigen and K2 capsule in ExPEC homeostasis, as well as virulence and pathogenesis. This study (and others) has shown that temperature can influence extracellular polysaccharide regulation, which ought to be considered when conducting host-range analyses, particularly when assessing the therapeutic use of phage.

Interestingly, this study also showed that the O6 antigen provides a steric barrier to T2 infection in shielding the receptor, OmpF. The implication of O6 and K2 in the resistance of CFT073 to T2 is not a unique phenomenon, as LPS and/or capsule serve as protective barriers to phage in several bacterial species, including *Klebsiella* and S*treptococcus* (Dunstan et al., 2021; Wilkinson and Holmes, 1979; Beerens et al., 2021). Moreover, capsule and O antigen have been shown to protect other ExPEC isolates from T-even phage infections. Strain Nissle 1917 (O6:K5:H1) displays resistance to T4 (another T-even phage, closely related to T2), with K5 and LPS specifically conferring the resistance to T4 (Soundararajan et al., 2019). Similarly, other research has shown that an *E. coli* K-12 clone expressing an O16 antigen becomes resistant to T4 and P1 phages (Broeker and Barbirz, 2017). The aforementioned studies indicate that, *in E. coli*, the roles of capsules and smooth LPS as a phage defence mechanism are conserved across several distinct *E. coli* isolates and may be a key evolutionary function for the extracellular polysaccharides, in addition to their obvious role in immune evasion. Due to the prevalence of specific O- and K- serogroups in ExPEC isolates (e.g., K1, K2, K5, K30, and K92 capsules and O1, O2, O4, O6, O7, O8, O16, O18, O25, and O75) O antigens (Ciesielczuk et al., 2016; Wijetunge et al., 2015; Goller and Seed, 2010), phage or phage-derived enzymes, which target these structures, are of interest as potential avenues for novel therapeutics.

A TA system was identified in CFT073 just upstream of region 1 of the capsule biosynthesis operon gene *kpsF*. This phenomenon is not unique to CFT073, as over 500 *E. coli* genomes were shown to have capsule genes and the TA system located proximal to one another. Interestingly, upon performing subsequent ‘blastn’ analysis, excluding *E. coli* hits, the TA system was also found to be present in six other bacterial genomes, namely *Shigella sonnei* (two hits), *Salmonella enterica* serovar Typhimurium (one hit), *Salmonella enterica* serovar Virchow (one hit), and *Salmonella enterica* serovar Newport (two hits). Thus, it can be deduced that the TA system explored in this study can be found exclusively in *Enterobacteria* but is predominantly associated with *E. coli.*

The *E. coli* genomes with c3681-2 TA system homologues were focused on for this study and were sequence typed, phylotyped, and serotyped using in silico typing software. The K100 strains possessed distances between the TA system and *kpsF* between 3,785 and 3,795 bp, indicating that, for K100 strains, the capsule genes and TA systems were co-acquired during a single horizontal gene transfer event, leading to conserved distances between the two systems. Similarly, other strains of capsule type K2, K1, K5, K14, K15, and K52 possessed distances between 1,367 and 1,384 bp, also indicating a co-acquisition of capsule and TA system genes. The aforementioned capsule types (K1, K2, K5, K15, K52, and K100) are commonly associated with extra-intestinal infections, as are many of the sequence types possessing the TA system, such as ST131, ST73, ST69, and ST393 (Chung et al., 2012; Miajlovic et al., 2016).

As with capsule types and sequence types, extra-intestinal *E. coli* isolates are associated with specific phylogroups. For example, in a published analysis of 152 *E. coli* strains, the majority of those classified as phylogroup A were found to be human commensals; extra-intestinal isolates were largely assigned to group B2 isolates, followed by group D, whilst strains isolated from animals were predominantly associated with phylogroup B1 (Waters et al., 2020). This research identified that, of the 502 assemblies with c3682 homologues, which were chosen as part of this study, 96% belonged to phylogroups that are correlated with extra-intestinal *E. coli* isolates, B2 (83%) and D (13%). Taken together, the bioinformatic analysis of the assemblies showed (for the most part) the colocation TA system and Group 2 capsule genes as was originally observed in CFT073, in addition to a correlation with sequence types, phylogroups, and capsule types, often identified in extra-intestinal infections (Dale and Woodford, 2015; Gordon et al., 2008). Further supporting the link between the TA system and ExPEC-associated isolates are the results depicted in Supplementary Figure S2 and Supplementary Table S5, wherein 40% of a collection of *E. coli* bloodstream isolates possess the TA system just upstream of the capsule gene cluster. Of the 40% with the TA system, most isolates possessed the capsule types encoded by the 502 assemblies used in this study, indicating perhaps a link between certain Group 2 capsules and the TA system, rather than all Group 2 capsules.

Interestingly, the TA system and capsule biosynthesis genes are present on a pathogenicity island (PAI) known as PAI-*pheV or* PAI-I in CFT073 (Samei et al., 2016). This PAI is 123 kb in size and spans from c3556 to downstream of *kpsM* (Guyer et al., 1998). Thus, in CFT073, the TA and capsule genes were, in fact, coacquired through a single horizontal gene transfer event. One study has suggested that the high virulence scores and volume of virulence factors and prevalence of PAIs within group B2 isolates are correlated with a genetic background that permits frequent horizontal gene transfer events, thus explaining the high frequency of the TA system in B2 strains found in this study (Escobar-Páramo et al., 2004). In fact, CFT073 encodes numerous insertion sequence elements and transposases, for example, only 4,800 bp downstream of *kpsM*, at c3703, a transposase for IS4 is encoded, which may have been involved in the acquisition of PAI-*pheV*. Moreover, c3684 (immediately downstream of the TA system and upstream of region 1 of capsule operon) is a transposase and may have been implicated in the recombination of the capsule genes, allowing for the emergence and dissemination of distinct capsule serotypes.

Upon evaluation of the local genome context (synteny) of several of the genomes, high variability within region 2 of the capsule gene cluster was found between strains of different capsule serotypes, while the TA system, as well as capsule clusters region 1 and region 3, are largely conserved across the different capsule types (Figure 3). Such synteny heavily suggests that, for the strains subject to context analysis, the TA systems and capsule genes were acquired on a similar PAI as the one in CFT073, PAI-*pheV*. Alternatively, portions of PAIs can be acquired, subject to recombination, or lost over time, and as such, it is possible that, in some of the assemblies analysed, portions of the 123 kb PAI-*pheV* are present rather than the full island (Samei et al., 2016). In the K100 strains, the genomic island also appears to be present, albeit having undergone further horizontal gene transfer or recombination following the introduction of the genomic island into the strains, as evidenced by the increased distance observed between the TA system and *kpsF* in K100 genomes resulting from the presence of two additional genes, an IS21-like transposase and helper ATPase.

A bioinformatic analysis of *E. coli* Group 3 capsules (closely related to Group 2) by Hong et al. (2023) demonstrated that the Group 3 region 2 gene capsular clusters are recombination hot spots, leading to significant variations and subsequent influences on capsular biosynthesis and structural evolution. The aforementioned study found that such variations in Group 3 region 2 clusters can arise via three mechanisms: (a) recombination within the region, (b) the capture of entire region two blocks into existing clusters based on flanking region (regions 1 and 3) sequence conservation, and (c) subsequent divergence due to genetic drift or selection pressure to overcome host immunity or phage predation (Hong et al., 2023). Such principles, in particular scenario (b), can be applied to the variation observed in the gene synteny of the 50 kb region in the select genomes spanning the TA system and capsule operon (Figure 3). In fact, the presence of transposase genes next to region 1 of the K100 capsule gene cluster, as well the IS element found in region 1 of the K15 genome and the transposase located after region 3 of the K2 genomes examined in Figure 3, provides evidence for scenario (b), wherein region two genes were acquired and recombined onto the chromosome following the acquisition of the PAI. The conserved sequence of the TA system (as well as regions 1 and 3 of the capsule clusters) implies that the TA system is of benefit to ExPEC, perhaps suggesting an evolutionary function for the association of these regions due to the conserved distance between the two systems and the high sequence identity in regions of synteny.

A study by Arredondo-Alonso et al. (2023), which explored the evolutionary history of the K1 capsule, showed that the Group 2 K1 capsule has emerged in four distinct clonal groups of ExPEC independently and that, in most instances (59% of genomes analysed), the K1 capsule is colocated with a PAI and type II secretion system, thus displaying conserved upstream and downstream synteny (Arredondo-Alonso et al., 2023). These findings correlate with the findings detailed in this study, whereby Group 2 capsule genes are acquired on a PAI common to ExPEC, albeit with the aforementioned PAI undergoing several recombination and subsequent horizontal gene transfer events following its introduction to *E. coli* genomes, giving rise to derivatives of the original PAI encoding distinct capsule serotypes. The presence (and conservation) of the TA system on the PAI could be explained by either (a) its potential interplay with the capsule under stress/ upon phage predation, or (b) through preserving or maintaining the presence of the capsule genes within the genome. The TA systems are often denoted as ‘selfish genes’, due to their ability to propagate and maintain themselves within bacterial populations, even at the expense of the host organism (LeRoux and Laub, 2022). A *Klebsiella pneumoniae* study, which involved the analysis of 259 clinically relevant genomes, showed that TA system genes often were significantly associated or co-located with virulence and AMR genes, implying that the association of virulence genes with TA systems provides a benefit to bacteria, in addition to maintaining the genes within pathogenic lineages (Horesh et al., 2020). Moreover, conserving the upstream and downstream synteny next to capsule gene clusters permits frequent allelic exchange, allowing for the diversification of capsule serotypes.

In summary, the association between Group 2 capsule genes (the predominant capsule group in ExPEC) and the AbiEi/AbiEii TA system was conserved across more than 500 *E. coli* genomes. The aforementioned TA system was shown to influence the susceptibility of strain CFT073 to several bacteriophages and also was shown to be co-transcribed with capsule genes. Moreover, it was shown that the K2 capsule and TA system appear to act in synergy with regard to CFT073 resistance to Bas 46, a novel interaction between two phage defence systems, which requires further exploration. The function of the TA system in ExPEC lineages, such as phage protection specific to CFT073, was not elucidated and is a limitation of this study. This study also showed that the K2 capsule can serve as a target or barrier to several phage. Perhaps, there is a synergy between the two systems, whereby the TA system is not required for phage protection when the capsule is being expressed, hence the importance of both capsule genes and TA system genes in proximity to one another. Alternatively, the TA system could maintain the presence of this common PAI in ExPEC lineages due to the importance of the capsule in immune evasion during infection, as well as during phage defence (Horesh et al., 2020; Miajlovic and Smith, 2014).

## Data Availability

The high-throughput sequencing data analyzed in this study are publicly available. This data can be found here: https://www.ebi.ac.uk/ena/browser/view/PRJEB44839. Oligonucleotide primers for mutagenesis and quantitative PCR were designed based on the assembled genome sequence of strain CFT073 (GenBank accession number: AE014075.1).
