# Supplementary material for: Escherichia coli Group 2 capsules and their interplay with bacteriophages
Source: Front Microbiol. 2025 Sep 18;16:1588121. doi: 10.3389/fmicb.2025.1588121 (PMC12488582; doi:10.3389/fmicb.2025.1588121)
Supplement: Supplementary file 1 [file Data_Sheet_1.DOCX]

**Supplementary file**

**Tables S1-S4, Figures S1-S2**

**Table S1 Strains used in this study**

| **Strain** | **Genotype/ Description** | **Selectable marker** | **Reference** |
| --- | --- | --- | --- |
| ***Escherichia coli*** |  |  |  |
|  |  |  |  |
| CFT073 Wild-type | Prototypic urosepsis isolate; O6:K2:H1 serotype | None | (1, 2) |
| CFT073 Δ*ksl* | *ksl::kan* | Kan^R^ | (3) |
| CFT073 Δ*ksl::FRT* | *ksl::FRT* | None | This study |
| CFT073 Δ*waaL* | *waaL::kan* | Kan^R^ | This study |
| CFT073 Δ*waaL::FRT* | *waaL::FRT* | None | This study |
| CFT073Δ*ksl ΔwaaL* | *ksl::gent, waaL::kan* | Gent^R^, Kan^R^ | This study |
| CFT073Δ*ksl::FRT ΔwaaL::FRT* | *ksl::FRT waaL::FRT* | None | This study |
| CFT073 Δ*wzy* | *wzy::kan* | Kan^R^ | This study |
| CFT073 Δ*wzy::FRT* | *wzy::FRT* | None | This study |
| CFT073 Δ*waaG* | *waaG::gent* | Gent^R^ | This study |
| CFT073 Δ*waaG::FRT* | *waaG::FRT* | None | This study |
| CFT073 ΔTA | C3681-c3682::*gent* | Gent^R^, | This study |
| CFT073 ΔTA Δ*ksl* | C3681-c3682::*gent; ksl::kan* | Gent^R^, Kan^R^ | This study |
|  |  |  |  |
| DH5α | K-12, *fhuA2 Δ(argF-lacZ)U169 phoA glnV44 Φ80 Δ(lacZ)M15 gyrA96 recA1 relA1 endA1 thi-1 hsdR17* | None | New England Biolabs |
|  |  |  |  |
|  |  |  |  |

Kan - Kanamycin, Gent – Gentamicin.

**Table S2 Plasmids used in this study.**

| **Plasmids** | **Description** | **Resistance** | **Reference** |
| --- | --- | --- | --- |
|  |  |  |  |
| pCP20 | Possesses FLP flip recombinase gene, 30°C temperature-sensitive replication | Amp^R^ | (4) |
| pKD4 | Possesses FRT-flanked kanamycin resistance cassette | Kan^R^ | (5) |
| pMH2 | Possesses gentamicin resistance cassette which was utilised in mutant construction | Gent^R^ | Hunt *et al.*, 2015, Unpublished |
| pKD46 | Used to construct mutants through homologous recombination, possesses λ-Red recombinase genes *exo bet* and *gam* which are induced by L-arabinose, temperature sensitive replication at 30°C | Amp^R^ | (5) |
| pBWB536 | Complementation of O6-antigen synthesis genes | Amp^R^ | (6) |
| pXLW36 | pWKS30 with *kslCDABE* for complementation of *ksl* mutant | Amp^R^ | (7) |
| pBAD/His-WaaG | pBAD backbone; Vector used for complementation of *waaG* mutations. Under control of P*araBAD* arabinose-inducible promoter | Amp^R^ | (8) |

Kan - Kanamycin, Gent - Gentamicin, Amp – Ampicillin.

| Table S3. List of oligonucleotides  Name | | Sequence (5’-3’) | | Purpose |
| --- | --- | --- | --- | --- |
| 1 | ATGTCGTTTTGTTGGAATGAAATTAACTCTGGTGTCAAGTCTTTAATTCTAT**TGTGTAGGCTGGAGCTGC** | **Amplify Kan^R^** cassette from pKD4 with homology to *waaL* | | |
| 2 | TTACTTATCTAATAAACATTGGTCCGATTGTACTTTAAAATAAGCACAAAGGTC**CATATGAATATCCTCC** | **Amplify Kan^R^** cassette from pKD4 with homology to *waaL* | | |
| 3 | GAGTCATTTGCGCACGAAAG | Screening primers for *waaL* in wild-type and mutant | | |
| 4 | AGATGGTTTGTAGGGCTCCG | Screening primers for *waaL* in wild-type and mutant | | |
| 5 | TAATGACGCAATTAAGTTATATCAAAATGATGAAAATGATGAAAATTTGAACATTTAGTATT**GTGTAGGCTGGAGCTGC** | **Amplify** **Kan^R^** cassette from pKD4 with homology to *kpsT* | | |
| 6 | GGGTATGAATAAAGATTTTTTGTTTGGATCAAAGTCAATATCATAATTAGGTC**CATATGAATATCCTCC** | **Amplify Kan^R^** cassette from pKD4 with homology to *kpsS* | | |
| 7 | GTCTTTATCAGAATATTAATGACGCAATTAAGTTATATCAAAATGATGAAAATTTGAACATTT**AGTGCGAATCCATGTGGGAGTTTA** | **Amplify Gentamicin^R^** cassette from pMH2 -homology to *kpsT* | | |
| 8 | GAATGCATTGGGTATGAATAAAGATTTTTTGTTTGGATCAAAGTCAATATCATAATTTA**TTAGGTGGCGGTACTTGGGT** | **Amplify Gentamicin^R^** cassette from pMH2 -homology to *kpsS* | | |
| 9 | CCCTGGTATGAAGCACGTTG | Screening primers for *ksl* operon in wild-type and mutant | | |
| 10 | CATGTCGTGGAGTTAAGCCG | Screening primers for *ksl* operon in wild-type and mutant | | |
| 11 | CGAATCCATGTGGGAGTTTA | Amplify Gentamicin^R^ cassette from pMH2 | | |
| 12 | TTAGGTGGCGGTACTTGGGT | Amplify Gentamicin^R^ cassette from pMH2 | | |
| 13 | TTGCCTTCCAGGCTGTTATC | *rplT* housekeeping control RT-PCR | | |
| 14 | CTGCTTTCGCTTTTTCAACC | *rplT* housekeeping control RT-PCR | | |
| 15 | CCCGTCATACTGACTGAGTACAT | *ksl2A* (region 2 capsule gene) RT-PCR | | |
| 16 | TGCGGTGATTTGCAGTATCC | *ksl2A* (region 2 capsule gene) RT-PCR | | |
| 19 | GCTCAGCAATAGCCTCGCCGCAATTGGCGTCGACAATATA**CGAATCCATGTGGGAGTTTA** | *waaG:****gent*** mutagenesis primers; homology to *waaG* | | |
| 20 | TCGATAAATTACTTCCCTCCTCCACGACAGGTACGTCGTT**TTAGGTGGCGGTACTTGGGT** | *waaG:****gent*** mutagenesis primers; homology to *waaG* | | |
| 21 | GCAATGAAGATTGCGTTAAC | *waaG* screen F | | |
| 22 | AGCGTGACCGAAATGAGATG | *waaG* screen R | | |
| 23 | CATATTTTTGCTATCATTGTGAAAATTTTTCGGAGGAAACT**GTGTAGGCTGGAGCTGCTC** | TA mutagenesis; **amplify gent cassette** | | |
| 24 | \| GGTCTGGTTCGATATGCTGAATTGTGGAATCGTACTTAACGCGTTTGCATATGAATATCCTCCTTA \| \| --- \| | TA mutagenesis; **amplify gent cassette** | | |
| 25 | CATAATTGCCAGGCACTATC | TA mutant screen F | | |
| 26 | GTTTTTGAGGCCACCAATGA | TA mutant screen R | | |

F; Forward, R; Reverse

| **Phage name** | **Other names (where relevant)** | **ICTV family** | **Morphotype** | **Reference** |
| --- | --- | --- | --- | --- |
|  |  |  |  |  |
| φEB-5 |  | *Drexlerviridae* | Siphovirus | (9) |
| φEB-32 |  | *Drexlerviridae* | Siphovirus | (9) |
| φEB-47 |  | *Drexlerviridae* | Siphovirus | (9) |
| φEB-49 |  | *Drexlerviridae* | Siphovirus | (9) |
| Bas04 | Fritz-Sarasin | *Drexlerviridae* | Siphovirus | (10) |
| Bas06 | KarlJaspers | *Drexlerviridae* | Siphovirus | (10) |
| Bas07 | JakobBernoulli | *Drexlerviridae* | Siphovirus | (10) |
| Bas09 | PaulSarasin | *Drexlerviridae* | Siphovirus | (10) |
| Bas32 | IrisVonRoten | *Demerecviridae* | Siphovirus | (10) |
| Bas35 | WilhelmHis | *Myoviridae* | Myovirus | (10) |
| Bas46 | ChristianSchoenbein | *Mosgiviridae* | Myovirus | (10) |

**Table S4.** List of Bacteriophage utilised in this study

**
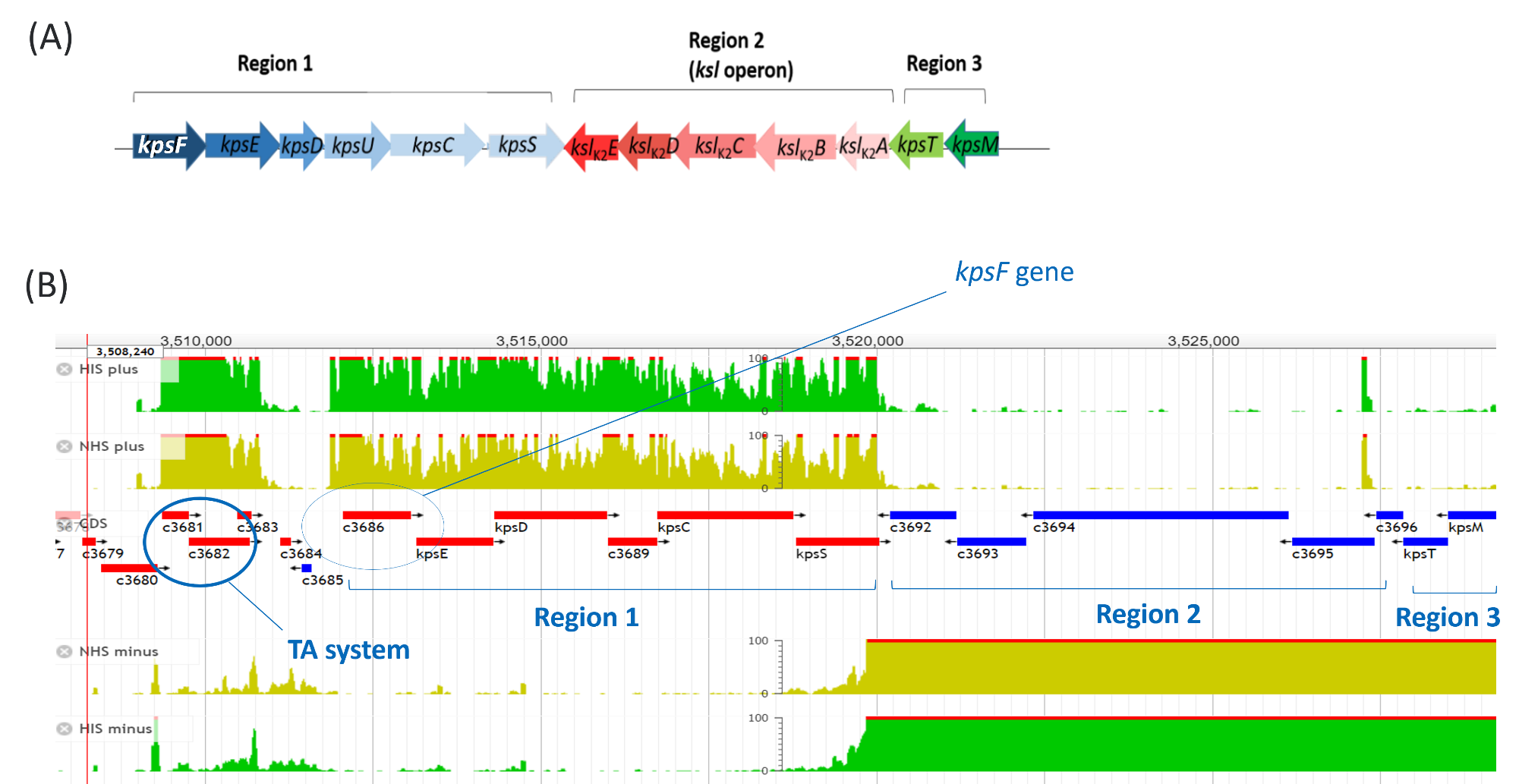
**

**Figure S1. TA system located just upstream of *kpsF*.** (A) Genetic organisation of the CFT073 K2 capsule. (B) The above image was taken from the JBrowse analysis function of RNAseq conducted by Miajlovic *et al* (3). Genes c3681 and 3682 are TA system genes.


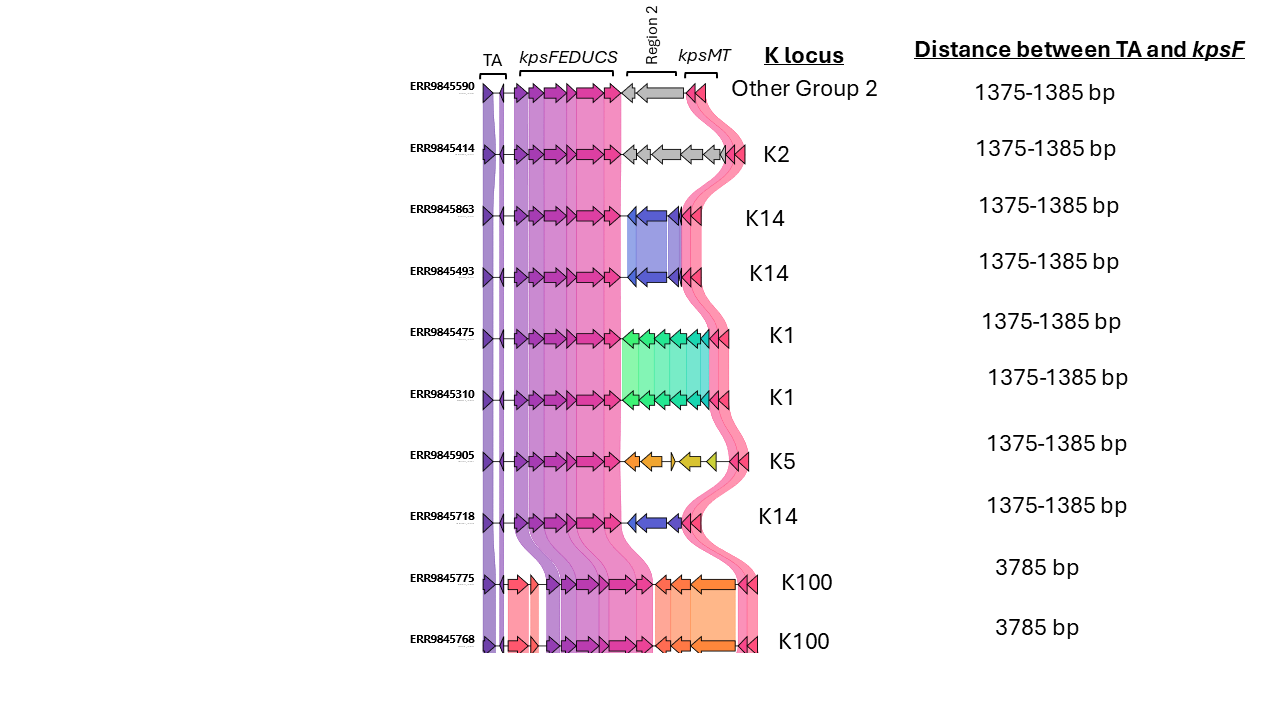


**Figure S2. Local context of the TA system in BSAC2 collection assemblies.** 10 random assemblies (out of 252) which possessed the TA system were analysed by Clinker to visualise gene synteny. As was seen in the original analysis, certain K loci are associated with distinct distances between the TA system and the capsule gene cluster, indicating both are frequently co-acquired even in distinct clonal groups of distinct capsule type.

References

1. Welch RA, Burland V, Plunkett G, Redford P, Roesch P, Rasko D, et al. Extensive mosaic structure revealed by the complete genome sequence of uropathogenic Escherichia coli. Proc Natl Acad Sci U S A. 2002;99(26):17020-4.

2. Guyer DM, Kao JS, Mobley HL. Genomic analysis of a pathogenicity island in uropathogenic Escherichia coli CFT073: distribution of homologous sequences among isolates from patients with pyelonephritis, cystitis, and Catheter-associated bacteriuria and from fecal samples. Infect Immun. 1998;66(9):4411-7.

3. Miajlovic H, Cooke NM, Moran GP, Rogers TR, Smith SG. Response of extraintestinal pathogenic Escherichia coli to human serum reveals a protective role for Rcs-regulated exopolysaccharide colanic acid. Infect Immun. 2014;82(1):298-305.

4. Cherepanov PP, Wackernagel W. Gene disruption in Escherichia coli: TcR and KmR cassettes with the option of Flp-catalyzed excision of the antibiotic-resistance determinant. Gene. 1995;158(1):9-14.

5. Datsenko KA, Wanner BL. One-step inactivation of chromosomal genes in Escherichia coli K-12 using PCR products. Proc Natl Acad Sci U S A. 2000;97(12):6640-5.

6. Sarkar S, Ulett GC, Totsika M, Phan MD, Schembri MA. Role of capsule and O antigen in the virulence of uropathogenic Escherichia coli. PLoS One. 2014;9(4):e94786.

7. Buckles EL, Wang X, Lane MC, Lockatell CV, Johnson DE, Rasko DA, et al. Role of the K2 capsule in Escherichia coli urinary tract infection and serum resistance. J Infect Dis. 2009;199(11):1689-97.

8. Muheim C, Bakali A, Engström O, Wieslander Å, Daley DO, Widmalm G. Identification of a Fragment-Based Scaffold that Inhibits the Glycosyltransferase WaaG from Escherichia coli. Antibiotics (Basel). 2016;5(1).

9. Battaglioli EJ, Baisa GA, Weeks AE, Schroll RA, Hryckowian AJ, Welch RA. Isolation of generalized transducing bacteriophages for uropathogenic strains of Escherichia coli. Appl Environ Microbiol. 2011;77(18):6630-5.

10. Maffei E, Shaidullina A, Burkolter M, Heyer Y, Estermann F, Druelle V, et al. Systematic exploration of Escherichia coli phage-host interactions with the BASEL phage collection. PLoS Biol. 2021;19(11):e3001424.
